# Acute stress alters transcript expression pattern and reduces processing of proBDNF to mature BDNF in *Dicentrarchus labrax*

**DOI:** 10.1186/1471-2202-11-4

**Published:** 2010-01-14

**Authors:** Chiara Tognoli, Federica Rossi, Francesco Di Cola, Gabriele Baj, Enrico Tongiorgi, Genciana Terova, Marco Saroglia, Giovanni Bernardini, Rosalba Gornati

**Affiliations:** 1 Department of Biotechnology and Molecular Science, University of Insubria, Varese-Italy; 2 Department for Life Sciences, BRAIN Centre for Neuroscience, University of Trieste, Trieste-Italy; 3 Centro di Ricerca Interuniversitario Politecnico di Milano e Università dell'Insubria "The Protein Factory", Italy

## Abstract

**Background:**

Stress involves alterations of brain functioning that may precipitate to mood disorders. The neurotrophin Brain Derived Neurotrophic Factor (BDNF) has recently been involved in stress-induced adaptation. BDNF is a key regulator of neuronal plasticity and adaptive processes. Regulation of BDNF is complex and may reflect not only stress-specific mechanisms but also hormonal and emotional responses. For this reason we used, as an animal model of stress, a fish whose brain organization is very similar to that of higher vertebrates, but is generally considered free of emotional reactions.

**Results:**

We provide a comprehensive characterization of BDNF gene in the *Dicentrarchus labrax *and its transcriptional, translational and post-translational regulation following acute stress. While total BDNF mRNA levels are unchanged, BDNF transcripts 1c and 1d resulted down regulated after acute stress. Acute stress induces also a significant increase in proBDNF levels and reduction in mature BDNF suggesting altered regulation of proBDNF proteolytic processing. Notably, we provide here the first evidence that fishes possess a simplified proteolytic regulation of BDNF since the pro28Kda form, generated by the SKI-1 protease in mammals, is absent in fishes because the cleavage site has first emerged in reptilians. Finally, we show that the proBDNF/totBDNF ratio is a highly predictive novel quantitative biomarker to detect stress in fishes with sensitivity = 100%, specificity = 87%, and Negative Predictive Value = 100%.

**Conclusion:**

The high predictivity of proBDNF/totBDNF ratio for stress in lower vertebrates indicates that processing of BDNF is a central mechanism in adaptation to stress and predicts that a similar regulation of pro/mature BDNF has likely been conserved throughout evolution of vertebrates from fish to man.

## Background

Brain Derived Neurotrophic Factor (BDNF) is the most abundant and widely expressed neurotrophin, a family of structurally related proteins required for the development and function of the vertebrate nervous system [1-3]. In the vertebrate brain, BDNF also governs long-lasting changes in synaptic efficacy and morphology [3-8]. Recent studies have suggested that BDNF may be involved in stress-induced adaptation in adult [9]. Indeed, several types of injury and cell stress affect the expression of BDNF in the mammalian brain; in particular, chronic stress decreases the synthesis of hippocampal BDNF [10-13] while acute stress induces complex alterations in the expression of BDNF, including a decrease in the hippocampus and an increase in the prefrontal cortex [14-17].

"Stress" is a biological term which refers to the consequences of the failure of a human or animal body to adequately answer to environmental stimuli. Stress induction is also used to study alterations of brain functioning leading to mood disorders which are often precipitated or exacerbated by acute or chronic stressful life events [18-20]. Of note, stress involves subjective feelings and experience of pleasure, pain, frustration, hunger or other states, and is therefore often difficult to define and measure [21]. This is particularly complicated in mammals and primates in which the emotional components may have a dominant effect. Since alterations in BDNF expression were also found in response to emotions such as anxiety or fear in rodents [22] and BDNF affects emotional preferences in humans [23], it remains to be determined how the stress itself or the associated behavioral responses contribute in mediating these changes. In this view, it is interesting to use as an animal model of stress, a fish whose brain organization is very similar to that of higher vertebrates, but is generally considered free of emotional reactions.

Stress conditions were traditionally evaluated by monitoring blood levels of cortisol, hemoglobin, and glucose [24], but these descriptors may not be sufficiently reliable when chronic stresses are applied and animal welfare is concerning [25]. It is therefore necessary to search for further parameters, which are capable to describe, taking into account the "allostatic concept" [26] biological stress and animal emotional responses. This point is particularly important when monitoring welfare of animals reared for commercial interest. In fact, production and quality have to be equally improved with benefits on the public perception of the products and consequent positive repercussions on marketing aspects. Accordingly, we started a search of alternative molecular biomarkers of stress. Besides the classical stress-related genes such as metallothioneins (MT), heat shock proteins (HSP), 3-hydroxil-3-methyl-glutaryl coenzymes A reductase (HMGCR) [27,28], enolase, Na^+^/H^+ ^exchanger *(NHE)-1 c-Fos*, glucocorticoid receptor (GR), glucose transporter (GLUT2) [29-32], plus genes related to specific stress conditions, as oxygen fluctuation (HIF-1α) and food deprivation [33-35], we decided to consider also neurobiological markers such as BDNF.

Accordingly, we investigated the effects of acute stress on BDNF expression in *Dicentrarchus labrax*. We report the genomic organization of *D. labrax *BDNF and its transcripts-specific expression pattern during post-hatching development. In addition, we analyzed BDNF expression in adult tissues, and post-translational processing in response to a well standardized type of acute stress such as water deprivation.

## Methods

### Animals

European seabass eggs, at stage of somites formation, were obtained from a commercial hatchery on Crete. The eggs were incubated in three 500 L-cylindriconical polyester tanks (~50,000 eggs/tank) at the Institute of Aquaculture of the Hellenic Center for Marine Research (Heraklion, Crete). During the autotrophic stages (complete absorption of lecith reserves) larvae were kept in darkness. The oxygen level was maintained at about 7 mg/L throughout the experimental period.

Following mouth opening and eye development, the larvae under intensive conditions are exposed to low light intensity (5-10 lux) without food for a period of 2-4 days until swim bladder is fully inflated. Only when inflated swim bladder is observed in more than 80% of the population, larvae were fed using an automatic feeding system. Ten larvae were taken every day for determining the morphological characteristic and total length.

The general conditions of rearing are presented in the Table 1, while the modality of the sampling, carried out in November 2008, are reported in Table 2. Pools of larvae for each developmental stage were randomly sampled and weighted. The samples were stored in RNAlater (Ambion, Austin, TX, USA) and kept at -20°C until the molecular biology analysis.

**Table 1:** conditions of rearing

	INTENSIVE
**Season of rearing**	**Winter - Spring**

Density of eggs	100 egg l^-1^

Water quality	Tank filled with filtered sea water from deep drill Renewal from biological filter Pseudogreen water method Closed recirculation system Controlled temperature and light

Temperature range	Constant 17,5 ± 1°C

Water renewal rate	Initially 10% h^-1^, gradual increase to 40% h^-1 ^at 35 dph

Photoperiod	12L:12D

Larval food	Enriched rotifers (5 ind ml^-1^), Enriched *Artemia *Instar II (0.5-1.0 ind ml^-1^) Artificial feed Daily addition of phytoplankton (in order to maintain a concentration of ca. 650 ± 300 × 10^3 ^cells ml^-1^) for 15 days after hatching


**Table 2:** sample timing

SAMPLES	dph	stage	total weight
**1a; 1b; 1c**	6	Mouth opened - black eyes	404 mg (wet)

**2a; 2b; 2c**	16	Lipid droplet absorption	300 mg (wet)

**3a; 3b; 3c**	27	Flexion	961 mg (wet)

**4a; 4b;4c**	33	Post-flexion	949 mg (wet)

**5a; 5b; 5c**	44	Dorsal and anal fins	1 g (wet)

**dph: **days post hatching

Fingerling seabass were obtained from Nuova Azzurro^® ^hatchery in Civitavecchia (RM, Italy), and reared into three fiberglass raceway tanks with 2.5 m^3 ^water each, with inconsistent mortality, at low biomass density (<10 Kg/m^3^). The tanks were connected to a water recirculation system where salinity (obtained adding salt Oceanfish 600 LT from Prodac Int^® ^to dechlorinated tap water) was 20 g/l. Other water conditions were: temperature 21 ± 1°C, pH 8.2, total ammonia <0.2 mg/l; dissolved oxygen was maintained over 99% of the saturation, by insufflating pure O_2 _to the system. At average weight of 450 g (adult animals), two groups of five animals were randomly sampled. The first group (control) was rapidly killed by severing the cervical column; brain, liver, kidney and muscle were removed, frozen in liquid N_2 _and stored at -80°C for molecular biology analysis. The second group (stressed) was kept for 30 minutes in a water deprivation condition (water volume of 20 L in a bucket 50 × 50 × 50 cm), then tissues were removed as described above.

The experimental protocol of this study was approved by the Ethics Committee of the University of Insubria

### Isolation and amplification of genomic DNA

Genomic DNA was extracted from 25 mg of liver with DNeasy Blood & Tissue Kit (Qiagen, Milan, Italy) according to the manufacturer's procedure.

Introns were amplified with primers designed in proximity of putative exon/intron junctions, inferred comparing ortologue sequences of BDNF (Genomic PCR section). All primers used in this paper are reported in Table 3. The PCRs were set using 500 ng of genomic DNA, Herculase Enhanced DNA Polymerase 5 U/μl (Stratagene, La Jolla, CA, USA) in its own buffer. The couple of primers used were Dl_ex1beta_fw and Dl_ex1a_rev, Dl_ex1a_fw and Dl_ex1c_rev, Dl_ex1c_fw and Dl_P2_ant_BDNF. The reactions were incubated in a thermal cycler at the conditions suggested in the manufacturer's procedure. PCR fragments were run on a 0,7% agarose gel, stained with ethidium bromide and run in TAE 1× buffer at 100 mV for 30 min. Single bands were gel-purified and sequenced.

**Table 3:** primers used in this paper

	NAME	SEQUENCE 5'-3'	Tm°C	Notes
Genome Walking	1beta_SP1	CCACTGAGTCCAACCCTTCCAGCAATGC	72.2	3
	1beta_SP2	ACCATTTTCCCCTACGCTGTCCTGGAGATAG	70.2	3
	1beta_SP3	ACTTCTGCTGTGCTCAGTAGATCGCCCACC	71.9	3
	1beta_SP4	GGCAAATATCAACAAGCCCGGGTTGTCAG	72.0	3
	1beta_SP5	GGCAATCCAAGTTTGTGGGGGTACTAGTTC	68.3	3
	1beta_SP6_A	GAGTGTTAACTCCCTCTTTGGCGAGGGG	69.7	3
	1beta_SP6_B	GGCCTATTACGCATACGCACAAACTGGTC	69.0	3
	AP1	GTAATACGACTCACTATAGGGC	59.0	4
	AP2	ACTATAGGGCACAGCGTGGT	71.0	4

Genomic PCR	Dl_ex1beta_fw	CCAAGTGGTGGGCGATC	57.6	2
	Dl_ex1c_fw	CCATGCAATTTCCACCATC	54.5	2
	Dl_ex1a_fw	GTTAACTTTGGGAAATGCAAG	54.0	2
	Dl_P1_ant_BDNF	CCATAGTAACGAACAGGATG	55.3	2
	Dl_P2_ant_BDNF	GTCATCACTCTTCTAACCTGTTG	58.9	2
	Dl_ex1a_rev	CACTTGCATTTCCCAAAGTTAAC	54.0	2
	Dl_ex1c_rev	GATGGTGGAAATTGCATGG	54.5	2
	Int1c/2_FW	CCAGACAGTTTCTGTATTGTTGTTTTGGAGGGG	70.2	3
	Int1c/2_REV	TCCAGCCATGTGAGGATCAATTGTGAACGG	73.6	3
	Int1c/2_up	GTATTTTCTTAATTGCACACAGCGTGGGTGGG	71.4	3
	Int1c/2_low	CATTCTTAATTGGTATCTGGGGCCGTGGC	71.0	3
	Int1c/2_FWnew	CTCTAGGTGCGTTGTCATGCACAAAGGC	69.8	3
	Int1c/2_REVnew	AGGGGTAATATTGCAGTAGCAGGGGGTGG	70.0	3
	Int1a/1c_FW	ATGCTCCCAATATGGGACCTTAAGACGCTGC	72.2	3
	Int1a/1c_FWnew	GTAATCGTTGCGTTGTGCTTAATCATGCTCC	66.8	3
	Int1a/1c_FW1	GGTCTGCTGCATTCATGTTTTGTCTTGATG	68.7	3
	Int1a/1c_FW2	GCCCTACTCTTTACCCCCCCCACCC	70.2	3
	1crev_SP1	TCTCCCGACAAGCTTCAGGATATCTCTTCAGC	70.8	3
	Int1a/1c_REV_1	TTTTGCGTAACIGCGCGTCTCCACCAIGTC	74.5	3
	Int1a/1c_REV_2	CAAACTCCTGGATATGAGCTTAAAGGAGGC	66.4	3
	Int1a/1c_REV_3	CGTTTGGCATGTAGCAGTATGGGAGTGG	69.3	3
	Int1a/1c_REV_4	CCTTTCAAGGCTTCTCTTGCCAAATGC	68.2	3
	Int1a/1c_REV_5	CATCCTGCCAGCATGTGCAACTGC	69.6	3
	Int1a/1c_REV_6	CCTGACTCACTTTTAGCCTATCTGACATGCAGG	69.3	3
	Int1a/1c_REV_7	CACACACACACACACACACACACACTGTG	68.3	3
	Int1beta_FW	GACCAGTTTGTGCGTATGCGTAATAGGCC	69.0	3

3'-RACE	SP1FW_3'	GGCTGCAGAGGAATAGACAAGCGGCAT	70.4	3
	SP2FW_3'	CCAATGCAGGACAACCCAGTCCTACGT	69.3	3
	BDNF_3'race	GACCATTAAGAGGGGCAGATAG	60.3	3
	AP	GGCCACGCGTCGACTAGTACTTTTTTTTTTTTTTTTT	71.1	4
	UAP	CUACUACUACUAGGCCACGCGTCGACTAGTAC	64.3	4

5'-RACE	RACE_BDNF_GSP1	CTTGGTTGCTGATCATC	50.4	3
	RACE_BDNF_GSP2	CTGTGAGTGAGGGCAGTTC	58.8	3
	RACE_BDNF_GSP3	CGAACAGGATGGTCATCACTC	59.8	3
	AAP	GGCCACGCGTCGACTAGTACGGGIIGGGIIGGGIIG	> 75.0	4
	AUAP	GGCCACGCGTCGACTAGTAC	66	4

RT-PCR	Dl_BDNF_up	ATGACCATCCTGTTCGTTAC	55.3	2
	Dl_BDNF_down	CTATCTGCCCCTCTTAATG	54.5	2
	1beta_FW_nested	GCGAGGGTGTTACGTATATCTG	58.7	3
	1beta_Rev_new	CCACTCACTCCAACAGATGC	59.3	3
	1a_FW_new	GCTTATTCTGAGGGAGCCTG	59.0	3
	1a_Rev_new	CCCAAAGTTAACGCAGTGTG	59.2	3
	1bFW	CTCAGCTCTGCAGAGTTGGGGT	61.8	3
	1c_FW_new	CGTTTCACCATGCGACAAC	61.1	3
	1c_Rev_new	GCCCAGTCGTAAAACAGACC	59.6	3
	1dFW	GTCCTGATGGAAACAGGAAATCAC	63.1	3
	1dRev	CACAGATGACGTCTCTTCCAGGT	62.9	3
	Ex2_FW_new	CTTCAGTTGCATGAGAGCTGC	61.3	3
	Ex2_Rev_new	ACCCTCATGCACATATTAGCG	60.0	3
	Dl_BDNFreal_low	TTGCTTCAGTTGGCCATTGG	57.3	3
	Dl_Act_FW_RT	GGTATTGTCATGGACTCCGGTGAT	61.9	1
	Dl_Act_Right	TTAGAAGCATTTGCGGTGGA	58.0	1
	D.l._GAPDH_FW	GAGGGTGACAAGCTGGTCGT	58.8	1
	D.l._GAPDH_Rev	CAAAGATGGAGGAGTGAGTGTCAC	58.8	1

Universal Primers	T7	TAATACGACTCACTATAGGG	53.2	
	SP6	CATTTAGGTGACACTATAG	50.2	

1 - primers deduced on sequences available in public databases (Actin: AY148350; GAPDH: AY863148). 2 - primers deduced on ortologue sequences (BDNF: Danio rerio AL935207 clone CH211-251J8; Fugu rubripes http://www.fugu-sg.org/ scaffold_1; Paralichthys olivaceus AY074888). 3 - primers deduced on obtained sequences. 4 - primers included in the kits used during the experiments.

Another set of PCRs on genomic DNA were performed with 80 nM solutions of specific primers deduced on obtained sequences, 250 ng of genomic DNA, 2 U of PCR Extender Polymerase Mix (5PRIME, Gaithersburg, MD, USA), 5 μl 10× Tuning Buffer, 0,5 mM dNTPs mix. The PCR was performed with the following conditions: 93°C for 3 min and 10 cycles at 93°C for 15 s, annealing at 65°C for 30 s elongation at 68°C for 8 min, plus 20 cycles with elongation time increased of 20 s each cycle. After gel electrophoresis single bands were gel-purified, cloned into pGEM-T Easy Vector (Promega, Milan, Italy), and sequenced.

### 5' Genome walking

To clone 5' flanking sequence of the gene, genome walking was carried out with the GenomeWalker Universal Kit (Clontech, Saint-Germain-en-Laye, France) according to the manufacturer's procedure. Briefly, aliquots of genomic DNA (2.5 μg) were separately digested overnight with the following blunt-end restriction endonucleases: *Dra*I, *Eco*RV, *Pvu*II and *Stu*I. After inactivation, the four digested DNA preparations were ligated to the GenomicWalker adaptors. Two rounds of PCR were performed with the BD Advantage 2 PCR kit (Clontech, Saint-Germain-en-Laye, France). Adaptor-ligated DNA fragments were used as template for primary PCR amplification, with the outer adaptor primer (AP1) and a gene specific 5'-outer primer (Table 3, Genome Walking section). Reactions were run using 0.2 μM solution of specific primers, 1 μl of template, 1 μl of 50× Advantage 2 Polymerase Mix, 5 μl 10× Advantage 2 PCR buffer, 0.2 mM dNTPs mix. The amplification protocol consisted of two-step cycle parameters: 7 cycles at 95°C for 25 s and 72°C for 3 min, 37 cycles at 94°C for 25 s and 67°C for 3 min plus a final extension at 67°C for 7 min. Aliquots (1 μl) of 50-fold diluted primary PCR products were used as template in the secondary PCR amplification, with the nested adaptor primer (AP2) and a nested gene-specific primer (Table 3, Genome Walking section) with the same reactions mix described above. The amplification protocol consisted of two-step cycle parameters: 5 cycles at 95°C for 25 s and 72°C for 3 min, 24 cycles at 94°C for 25 s and 67°C for 3 min plus a final extension at 67°C for 7 min. Amplified products were analyzed in 1% agarose gel and sequenced as above reported.

### Endonucleases digestion

The 3 Kb PCR product obtained with the primers Int1a/1c_Fwnew and Int1a/1c_REV_5 was very tricky in cloning and sequencing steps because of the presence of highly repeated region. For these reasons a blunt digestion, with 1 U of *Hae*III/1 μg of PCR product, was performed in order to obtain smaller fragments. The reaction was incubated at 37°C for 2 h. The four bands obtained from the digestion, of 1.5 Kb, 1 Kb, 0.4 Kb, 0.1 Kb respectively, were gel purified, A-tailed with DNA Polymerase, ligated into pGEM-T Easy Vector (Promega, Milan, Italy), and sequenced.

### RNA extraction, mRNA retro transcription and amplification

Total RNA was extracted with TRIzol Reagent (Invitrogen, S. Giuliano Milanese, MI, Italy) from about 100 mg of each pool of larvae and tissue following the manufacture's instruction, then treated with DNase (DNA free, Ambion, Austin, TX, USA). The first strand cDNA was synthesized using 2 μg of total RNA, 150 pmol random primers (for larvae's RNA) and dT16 primer (for tissues' RNA), 1 μl dNTPs mix 10 mM, in a volume of 12 μl. The mix was heated at 65°C for 15 min, chilled on ice and then 4 μl 5× retrotranscription buffer, 2 μl of 0.1 M DTT, 1 μl RNaseOUT and 200 U M-MLV retrotranscriptase (Invitrogen, S. Giuliano Milanese, MI, Italy) were added to a final volume of 20 μl. After incubation at 37°C for 50 min, the reaction was stopped at 75°C for 15 min. The generated cDNA was stored at -20°C.

The open reading frame was obtained by RT-PCR performed with specific primers Dl_BDNF_up and Dl_BDNF_down designed within conserved regions of BDNF coding sequence belonging to other species. The bipartite BDNF transcripts were evaluated, when necessary, by two rounds of PCR with primers deduced on the obtained exon sequences. Reactions were run using 1 μM solution of specific primers (Table 1, Qualitative PCR section), 1 μl of cDNA, 0.75 U of GoTaq DNA Polymease (Promega, Milan, Italy), 5 μl 5× Green GoTaq Reaction buffer, 0.2 mM dNTPs mix. The first round PCR was performed with the following conditions: 94°C for 3 min and 34 cycles at 94°C for 30 s, annealing at 56°C for 30 s, elongation at 72°C for 50 s and final extension at 72°C for 4 min. The second round PCR was performed on 1 μl of first round PCR product for 30 cycles at the same conditions.

The PCR products were loaded into 1% agarose gel stained with ethidium bromide and run in TAE 1× buffer at 100 mV for 30 min; β-actin and GAPDH were used as housekeeping genes. For each sample a set of PCR has been run without retrotranscription to exclude any genomic contamination.

### 5' and 3' Rapid Amplification of cDNA Ends (RACE)

The 5'-RACE was performed according to the method published by Semple-Rowland et al., [36] with slight modifications. Briefly, 1 mg poly-A^+ ^RNA, extracted from seabass brain, was reversed transcribed using 200 U M-MLV reverse transcriptase (Invitrogen, S. Giuliano Milanese, MI, Italy) following the manufactured instruction and using 20 pmol of sequence-specific antisense primer RACE_BDNF_GSP1. The reaction was incubated at 42°C for 50 min and stopped placing the tube on ice; excess primers, dNTPs and buffer were removed using a QIAquick PCR purification kit (Qiagen, Milan, Italy). In the final step of the procedure the DNA was eluted in 30 ml of water. A poly dCTP tail was added to the single-stranded cDNA present using terminal deoxynucleotidyl transferase (Promega, Milan, italy). The mixture was denaturated at 94°C for 3 min, chilled on ice, incubated at 37°C for 10 min and stopped at 70°C for 10 min; excess of dCTP and buffer was removed as reported above. Second strand cDNA synthesis was carried out using 5 U TaqPolymerase (Qiagen, Milan, Italy), 0.2 μM of a poly d(G) anchor primer (RACE_AAP), 200 mM dNTPs mix and 10× PCR buffer. The reaction was incubated in a thermocycler at the following conditions: 40°C for 5 min, 72°C for 2 min, than the temperature was increased at 80°C. At this point 0.2 mM of the nested sequence-specific primer RACE_BDNF_GSP2 and a nested anchor primer RACE_AUAP were added for the amplification at the following conditions: 94°C for 1 min, 54°C for 1 min, 72°C for 1 min (30 cycles), last extension time 72°C for 10 min; kept at 4°C. 1 ml of a 1:10 dilution of the PCR products is re-amplified using the nested anchor primer RACE_AUAP and the nested sequence-specific primer RACE_BDNF_GSP3. The PCR cycle parameters were as follow: 8 touchdown cycles with annealing temperature from 58 to 54°C, than 94°C for 1 min, 54°C for 1 min, 72°C for 1 min (27 cycles), last extension time 72°C for 10 min; kept at 4°C. The resulting products were run on a 1% agarose gel, purified, cloned into pGEM-T Easy Vector (Promega, Milan, Italy) and sequenced.

The 3' race was performed with the following protocol: 4 μg of total RNA and 10 pmol of Adapter Primer (AP) in a volume of 10 μl was incubated at 70°C for 10 min. The mix was chilled on ice and then 4 μl of 5× reverse transcription buffer, 2 μl of 25 mM MgCl_2 _solution, 1 μl of 10 mM dNTPs mix and 2 μl of 0.1 M DTT were added. The mix was incubated at 42°C for 5 min and then 200 U of SuperScript III reverse transcriptase (Invitrogen, S. Giuliano Milanese, Italy) were added. After incubation at 42°C for 50 min, the reaction was stopped at 70°C for 15 min. The generated cDNA (2 μl) was used as template for PCR. The reactions were run using 1 μM solution of Universal Amplification Primer (UAP) and gene specific 3'-outer primer (SP1FW_3'), 0.75 U of GoTaq DNA Polymease (Promega, Milan. Italy), 10 μl 5× Green GoTaq Reaction buffer, 0.2 mM dNTPs mix. The reaction was incubated in a thermocycler at the following conditions: 95°C for 2' and 30 cycles at 95°C for 30 s, annealing depending on the melting temperature of the primers for 30 min, elongation at 72°C for 2 min and final extension at 72°C for 6 min. A second round PCR was performed at the same conditions using 1 μl of first PCR product, 1 μM solution of UAP and a nested gene-specific primer (SP2FW_3', BDNF_3'race). The resulting products were run on a 1% agarose gel, purified, cloned into pGEM-T Easy Vector (Promega, Milan, italy) and sequenced.

### Semiquantitative analysis

The bipartite BDNF transcripts expression, in control and stressed brain samples, were evaluated by semiquantitative PCR. The reactions were performed with the same specific primers and conditions of qualitative PCR, and normalization was carried out using cytoplasmatic β-actin (cDNA 1:50 diluted). The PCR products were loaded into 1% agarose gel and run in TAE 1× buffer at 100 mV for 30 min. The semiquantitative analysis was carefully performed by BIO-RAD Gel Doc 2000 connected to the software Quantity one™ that allowed to determine, in arbitrary units, the fluorescence value of the area of each considered band. After having obtained all the values, we have normalized them with those of β-actin; than we evaluated the ratio of the "stressed" samples compared to the control ones. In this way, we have avoided differences due to template concentration in the PCR tube. The data were statistically compared using the two tail omoschedastic Student's *t*-test. The significance level was set at *p *< 0.05.

### Bioinformatic analysis

BDNF gene exon-intron boundaries were determined by Blast, ClustalW analysis http://blast.ncbi.nlm.nih.gov/Blast.cgi; http://www.ebi.ac.uk/Tools/clustalw2/index.html and by direct comparison of PCR-amplified sequences with genomic pufferfish http://www.fugu-sg.org/BLAST/Export.htm, zebrafish, human and rodent DNA from the NCBI database (GeneBank accession number: AL935207 clone CH211-251J8; AF411339; AY057907, respectively).

### Western-blot analysis

Brain and liver were extracted from control (N = 15) or stressed animals (N = 15) and immediately frozen in liquid nitrogen. The tissues were mechanically homogenized at 4°C using an extraction buffer solution containing 25 mM Tris HCl ph 7.5, EDTA 1 mM, Spermidin 1 mM, PMSF 1 mM, IAA 1 mM, Soy Bean Trypsin Inhibitor (SBTI), 10 μg/ml Turkey Egg White inhibitor (TEWI). After homogenization 0.1%Triton X-100 was added and samples were incubated in agitation for 1 hour at 4°C. Samples were centrifuged for 5 min, at 4°C (10.000 × g) and the soluble fraction (supernatant) of the lysate was collected for Western blot analysis. Total protein content in lysate tissue samples was determined using Bradford assay (Sigma-Aldrich). Samples (10 μg) were run in 15% SDS-PAGE and proteins were transferred onto a nitrocellulose membrane (Protran Nitrocellulose Transfer Membrane, Whatman) using transfer buffer solution [39 mM Glycine, 48 mM Tris-HCl, 0,037% (v/v) SDS, 20% (v/v) methanol]. Subsequently, the membrane was stained using Ponceau dye (Sigma-Aldrich) to check for the complete protein transfer. Membranes were cut at the level of 44 kDa according to protein markers. The two membranes were incubated for 1 hour at room temperature in blocking solution (4% (v/v) non fat milk powder, 0.05% Tween-20 in phosphate buffer saline solution). The upper part of the membrane (>44 kDa) was incubated over night (O/N) at 4°C with anti-α-tubulin antibody (Sigma-Aldrich, mAB diluited 1:10.000). The lower part of the membrane (<44 kDa) was incubated with anti-BDNF antibody (N-20, pAB, Santa Cruz Biotechnology, diluted 1:500). The anti-BDNF antibody recognizes the first 20 N-terminal aminoacids of mature BDNF and therefore is able to detect both the mature and the precursor form of BDNF. Moreover, as the human and seabass mature BDNF are highly homolog (more than 90%) we have used human BDNF as positive control [37,38]. After O/N hybridization with the specific antibody, membranes were incubated with secondary antibodies for 1 hour at room temperature, we used goat anti-mouse HRP (Sigma-Aldrich, dil. 1:20.000) for α-tubulin, and goat anti rabbit HRP (DakoCytomation, dil. 1:10.000) for BDNF. Finally, membranes were washed with blocking solution and immunoreactive bands were detected using a chemiluminescence system (ECL-advance, Amersham Biosciences).

### Densitometry and statistical analysis

Densitometric analysis of immunoreactive bands was obtained by scanning films at 16-bit level and applying Quantity One software procedures (Biorad). Data were normalized using as internal control the Western blot for the housekeeping gene α-tubulin. The ratio ProBDNF vs total-BDNF or matBDNF vs total-BDNF was expressed as % and obtained with the formula: proBDNF/(proBDNF+matureBDNF)×100. Each set of data was statistically analyzed using Student's *t*-test and one-way ANOVA (Holm-Sidak). The statistical analysis was performed using SigmaStat 3.1 software. A p value of 0.05 was set as the minimal level for statistical significance.

### Calculation of test performance

We considered positive to the proBDNF/totalBDNF test, individuals whose score was >1SD with respect to the average value in the normal, non-stressed population. Stressed animals positive to test are true positive (= a), non-stressed animals which tested positive are false positive (= b), stressed animals that tested negative are false negative (= c) while non-stressed animals that tested negative are true negatives (= d). The sensitivity, calculated as a/(a+c), measures the proportion of actual positives which are correctly identified as such; and the specificity, calculated as d/(d+b), measures the proportion of negatives which are correctly identified. The positive predictive value is the probability that a test positive is a true positive: a/(a+b) and it is the most important measure of a diagnostic method as it reflects the probability that a positive test reflects the underlying condition being tested for. Its value does however depend on the prevalence of the disease, which may vary. The negative predictive value is the probability that a test negative is a true negative: d/(c+d). The negative predictive value is the proportion of individuals with negative test results who are correctly identified.

## Results

### Genomic organization of BDNF

As the gene encoding BDNF in *Dicentrarchus labrax (D. labrax*) was not described before, we first cloned the entire gene and determined its genomic organization. We used a strategy of cloning each exon separately using PCR primers designed on a consensus sequence inferred from the ortologue sequences of BDNF in *Danio rerio *(zebrafish), and *Fugu rubripes *(pufferfish). Zebrafish, pufferfish, and seabass are all teleosts and therefore we expected a similar exon/intron organization of their BDNF gene and closely related sequences. To clone the 5' flanking sequence of the *D. labrax *BDNF gene, we carried out a genome walking. Finally, to determine the *D. labrax *BDNF gene exon/intron boundaries and identify the mRNAs transcribed from the gene, we performed a combination of 5' and 3' rapid amplification of cDNA ends (5' and 3' RACE), RT-PCR and bioinformatic analysis.

**Figure 1:**
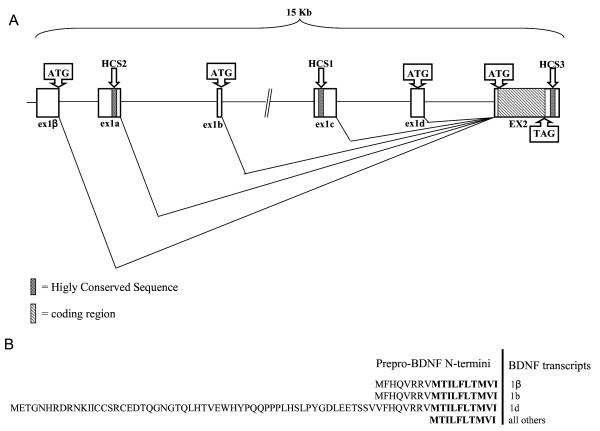
**Gene organization and amino acid sequence of BDNF**. **Panel A**. Organization of BDNF gene in *Dicentrarchus labrax*. Exons are shown as boxes and introns as lines. Alternative start codons (ATG) as well as stop codon (TAG) were reported. **Panel B**. Amino acid sequences of different potential prepro-BDNF N-termini. Amino acids encoded by exon 2 are in bold and sequences encoded by alternative in frame ATG are in normal style. The transcripts encoding the respective N-termini of BDNF (i.e., 1β, 1b, 1d) are listed adjacent to the N-terminal sequences.

The gene spans about 15 Kb and it is organized in 6 exons and 5 introns as reported in Fig. 1A (GeneBank accession number FJ711591). Exons were identified by ClustalW analysis (see methods) as the most highly conserved segments and were all found to be flanked by the typical consensus splice donor (GT) site in eukaryotes. The exons length and position, and their exon/intron junctions are summarized in Table 4. In analogy with zebrafish and pufferfish, also in *D. labrax *the BDNF coding sequence is contained in the exon 2 and this tract resulted highly conserved with respect to other vertebrate species (*D. rerio *84%, *F. rubripes *91%, *H. sapiens *77%, *M. musculus *78%, *R. norvegicus *78%, ). Upstream to the coding exon we have found other five untranslated exons: 1β, 1a, 1b, 1c and 1d. By aligning these exon sequences with those of the corresponding zebrafish exons, we found an identity of 85%, 43%; 82%; 74% and 82%, respectively. *D. labrax *BDNF transcripts analysis indicated that upstream untranslated exons can be spliced independently to the major coding exon to form distinct bipartite BDNF transcripts with different 5' UTR lengths and a common coding region (GeneBank accession number DQ915807). Interestingly, in the exons 1d, 1b and 1β we have identified in-frame ATG codons that could be used as translation start sites leading to the prepro-BDNF proteins with longer N-termini (Fig. 1B).

**Table 4:** Structure of *Dicentrarchus labrax *gene*

Exon	Start	Splice acceptor	End	Splice donor	Length (bp)
**1β**	nt 321	-	nt 602	GGAAAATG**gt**aagtag	282

**1a**	nt 1367	-	nt 1818	TTGTAAAG**gt**aagagc	452

**1b**	nt 4270	-	nt 4315	ACCTGATG**gt**aggttt	46

**1c**	nt 8347	-	nt 8646	AGTAAAAG**gt**atgtgt	300

**1d**	nt 11540	-	nt 11797	CTGTGGTT**gt**tatgct	258

**2**	nt 14063	ccctcc**ag**TTCCACCA	nt 15130	-	1068

* Position numbering is based on gene sequence

**Figure 2:**
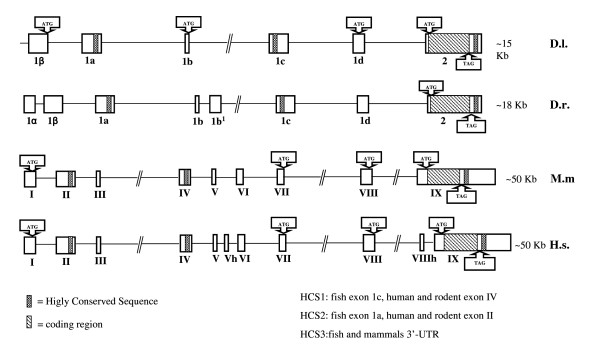
**Organization of BDNF gene in man, rodent and fish**. Exons are shown as boxes and introns as lines. D.l.: seabass BDNF; D.r.: zebrafish BDNF; M.m.: rodent BDNF; H.s.: human BDNF.

**Figure 3:**
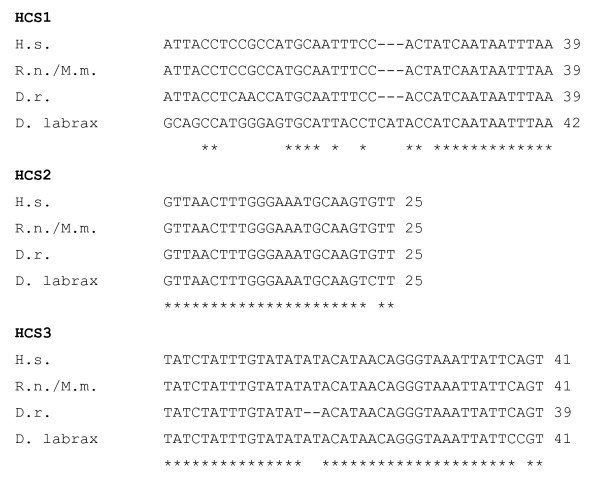
**Alignment of Highly Conserved Sequence in man, rodent and fish**. (H.s) *Homo sapiens*, (R.n.) *Rattus norvegicus*, (D.r.) *Danio rerio*, (D.l.) *Dicentrarchus labrax*.

The five exons located upstream to the coding region did not show any significant identity when aligned with mammalian BDNF genes (rat, mouse and human) with the exception of a 75% identity between *D. labrax *exon 1β and mammalian exon 1, and for the presence of the highly conserved segments HCS1, HCS2 and HCS3. HCS2 is located in *D. labrax *BDNF exon 1a and mammalian exon IIC and showed 96% identity; HCS1 in *D. labrax *BDNF exon 1c showed 38-41% identity with a similar sequence in mouse, rat and human exon IV while the HCS3 is localized in the 3'UTR of *D. labrax*, mouse, rat and human BDNF and was 97% identical (39 of 41 nucleotides are identical in fish and man) between these species (Fig. 2 and 3).

**Figure 4:**
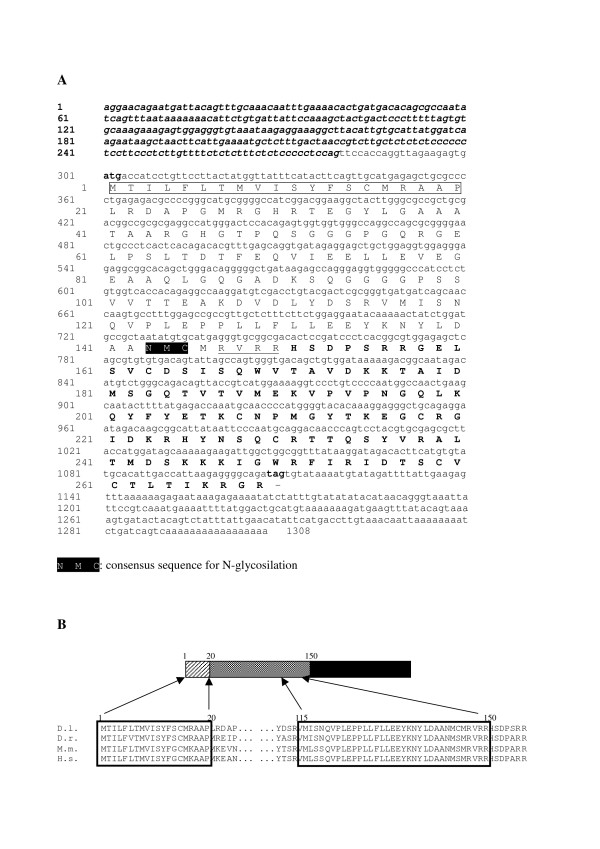
**Protein and cDNA sequence of BDNF**. **Panel A**. Dicentrarchus labrax cDNA sequence and deduced amino acid sequence (GeneBank accession number genFJ711591FJ711591). AA sequence of signal peptide is boxed in white; AA sequence for N-glycosilation is boxed in black; The cleavage sequence is underline; start codon and the mature BDNF are shown in bold. **Panel B**. Representation of the entire BDNF protein and alignment, among different species, of AA sequence of two conserved regions in the prepro protein: *Dicentrarchus labrax *(D.l.); *Danio rerio *(D.r.); M.m. *Mus musculus*; *Homo sapiens *(H.s). The dashed, the stippled and the black areas correspond to the signal peptide, the propeptide and mature secreted protein respectively.

The coding region encoded a protein precursor (Fig. 4A) with a signal peptide at the N-terminus, the propeptide of 150 amino acids (AA) in the center and the mature BDNF of 129 amino acids at the C-terminus. This organization is similar to that of zebrafish [39] avian [40] and mammalian BDNF [41-43]. The proBDNF resulted only 87% identical to zebrafish BDNF and 74-75% to the mammalian counterparts. However, two regions were >95% identical (Fig. 4B): the first 20 N-terminus AA, comprising the signal peptide, and 35 AA just upstream of the cleavage site which also encoded for the glycosilation consensus site (Fig. 4A). Analysis of the extended N-terminal sequences with the prediction programme SignalP 3.0 [44] showed that the N-termini produced by exons 1β and 1b have poor scores as signal peptides because of the presence of a putative signal anchor, while the very long sequence produced by exon 1d does not encode for a signal peptide.

### Developmental and tissue-specific expression of BDNF splice variants

**Figure 5:**
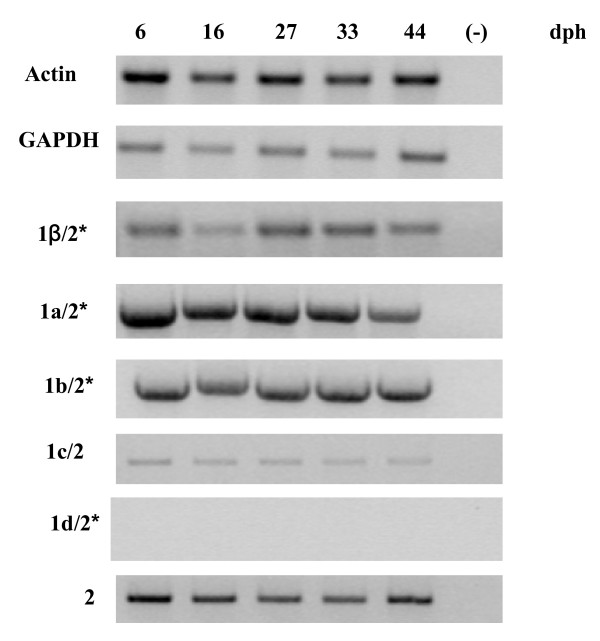
**Example of expression of *Dicentrarchus labrax *alternative mRNAs during larva development obtained by RT-PCR**. *: Aliquots of first PCR products were amplified in 2nd round of PCR. For details see Material and Methods.

To learn more about the possible role of BDNF transcripts in the seabass, we analyzed their expression during post-hatching development and their tissue distribution in the adult. The different transcripts were amplified using 5' exon forward specific primers in combination with a reverse primer located on the exon 2. Expression of the coding exon 2 was determined using internal primers. When no amplicon was detectable after the first PCR reaction, a second round of PCR was carried out to increase sensitivity. Analysis of BDNF expression at 6, 16, 27, 33 and 44 days post-hatching (dph) showed that, besides variant 1d/2, all BDNF variants were expressed during the entire larval maturation. Of note, variant 1d/2 transcript was undetectable at all stages even after the second round PCR. Although this analysis cannot be considered quantitative, it is clear that the generated bipartite transcripts showed striking differences in their expression with 1c/2 splice variant showing the highest expression throughout all post-hatching development stages (Fig. 5).

**Figure 6:**
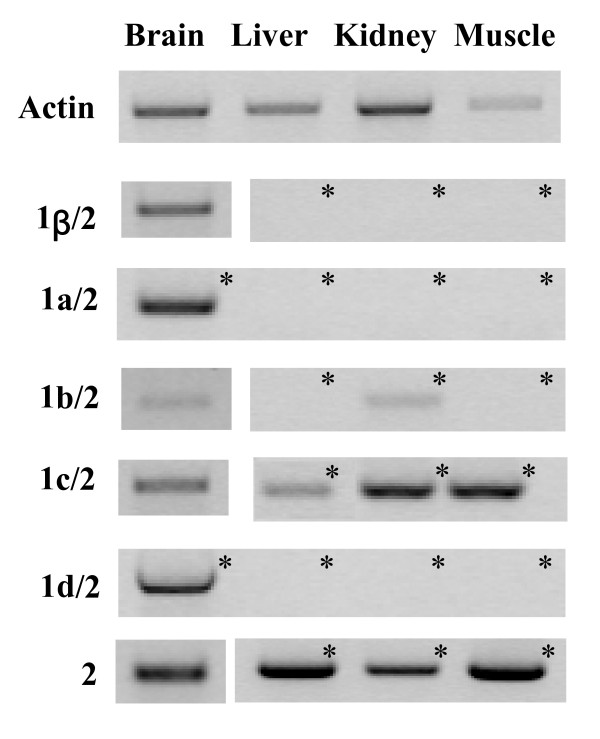
**Example of expression of *Dicentrarchus labrax *alternative mRNAs in different tissues of adult animals obtained by RT-PCR**. *: Aliquots of first PCR products were amplified in 2nd round of PCR. For details see Material and Methods.

Expression of splice variants was also determined in brain, liver, kidney and muscle of adult animals. An example of the PCR analysis, after gel electrophoresis, is shown in Fig. 6. The highest expression levels of the *D. labrax *BDNF transcripts were observed in the brain even though some variants, such as 1b/2 and 1c/2, were detected also in non neuronal tissues even if only after a second round of PCR. A semi-quantitative evaluation of the tissue-specific expression of *D. labrax *BDNF alternative transcripts is reported in Table 5. Bioinformatic promoter analysis using Transfac^® ^6.0 and public version of Match™ software [45,46] highlighted two regions (TGACGTCA and TGAAGTCA), upstream to the exon 1c with highly conserved consensus for the cAMP/calcium responsive element binding protein (CRE) which are also found in mammals upstream to the exon IV [47-50]. The presence of the HCS1 in fish exon 1c and mammalian exon IV supports the likelihood that these two exons are true orthologs.

**Table 5:** Expression of 5' non coding and coding exons in different tissues of adult *D. labrax*

EXON	BRAIN	LIVER*	KIDNEY*	MUSCLE*
1β/2	+	n.d.	n.d.	n.d.

1a/2	+*	n.d.	n.d.	n.d.

1b/2	+/-	n.d.	+/-	n.d.

1c/2	+	+/-	+	+

1d/2	+*	n.d.	n.d.	n.d.

2	++	+	+	+

* An aliquot of first PCR product was amplified in a 2nd round of PCR

**n.d.: **not detectable

### Effects of acute stress on the expression of BDNF splice variants

**Figure 7:**
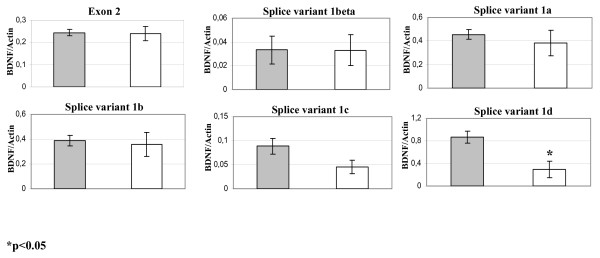
**Histogram of semiquantitative-PCR of BDNF**. Variants and coding exon in control are reported as grey bars, stressed samples are reported as white bars. Values are means ± SD; *p < 0.05. n = 5.

Since acute stress induces variations in BDNF transcripts expression in the brain of rodents [14,17,15] we investigated if any changes occurs when fishes underwent to a brief stressful event consisting in 30 minutes of controlled water deprivation condition (see methods). Semi-quantitative PCR analysis of BDNF transcripts expression in the seabass brain revealed that in the stressed group there were no significant differences in the expression of coding exon 2, and in the upstream exons 1β, 1a, 1b compared to the control group (Fig. 7). In contrast, we found a significant decrease in the expression of the exons 1c and 1d (p < 0.05; Fig. 7). Thus, acute stress in the seabass, in absence of an emotional component, induces a rapid down regulation of the exons belonging to the second exon cluster.

### BDNF protein processing in seabass after acute stress

**Figure 8:**
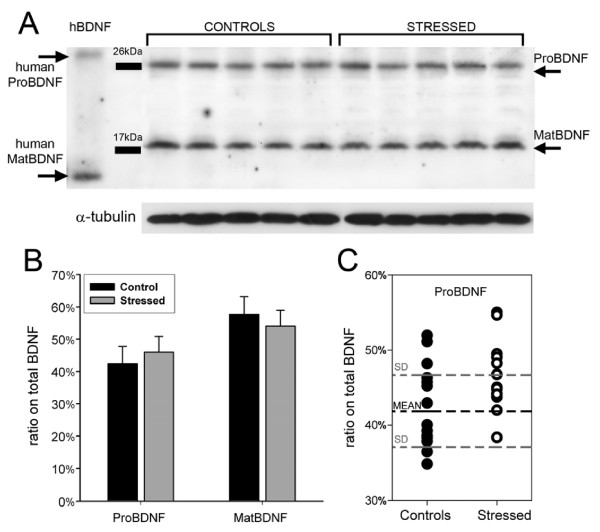
**Stress does not alter the ratio of pro/matBDNF in the seabass liver**. A) A representative Western blot of proBDNF and matBDNF expression in the seabass liver. Alpha-tubulin (MW 55 KDa) is used for normalization. Human recombinant proBDNF and matBDNF shows reactivity of the anti-BDNF antibody. The bands corresponding to the seabass proBDNF and matBDNF are indicated on the right side. B) Mean percentage of proBDNF vs. total BDNF (totalBDNF = proBDNF+matBDNF) of 10 control and 10 stressed animals. Error bars represent SE. No significant difference between control and stressed animals was found. C) Scatter-plot showing the large overlap of the percentage of proBDNF on total BDNF in individual animals from the control and the stressed populations. The F values, obtained by One way Anova analysis, for proBDNF and mature BDNF were 4.154 and 0.055, respectively.

**Figure 9:**
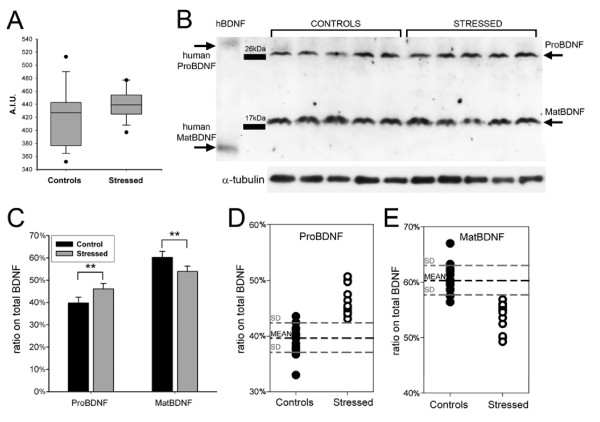
**Stress increases significantly the percentage of proBDNF while decreasing matBDNF in the brain of stressed seabass**. A) The quantification of total BDNF in the brain of seabass using an ELISA assay, showed no difference between control and stressed group (A.I.U. = arbitrary intensity units). B) A representative Western blot of proBDNF and mature BDNF expression in the seabass brain. Human proBDNF and mature BDNF is shown in the first lane from left. Alpha-tubulin used for normalization is shown at the bottom. C) ProBDNF on total BDNF is significantly increased (** = p < 0.01) in stressed animals with respect to control animals and mature BDNF percentage is increased (** = p < 0.01; n = 15 animals, each in duplicate. Error bars are SE). D) The scatterplot shows that in the brain of all stressed animals proBDNF are above the mean of controls+1SD. E) Similarly, in all stressed animals mature BDNF values are below the mean of controls -1SD. Only 2 control animals have a proBDNF percentage above the controls' mean+1SD for proBDNF or below the mean-1SD. The F values, obtained by One way Anova analysis, for proBDNF and mature BDNF were 19.028 and 6.225, respectively.

To understand if acute stress can also alter BDNF protein levels and/or processing, we analysed by Western blot the liver and the brain from 15 normally reared controls and 15 animals that underwent acute stress. In both liver and brain, anti-BDNF antibodies recognized two bands with apparent molecular weight of 27 KDa and 18 KDa which correspond to proBDNF (calculated Mw = 28,7 KDa) and mature BDNF (calculated Mw = 13,3 KDa), respectively (Fig. 8A and 9A). Remarkably, seabass BDNF does not contain the cleavage site (RGLT) that in mammals is recognized by the Membrane-bound transcription factor site-1 protease (MBTPS1 also known as SKI-1 protease) to generate the pro28KDa-BDNFisoforms after a cleavage at Threonin 57 [51]. Thus, in fishes, there is only a proBDNF (equivalent to mammalian pro32KDa) and a mature BDNF.

Preincubation of the anti-BDNF antibody with the corresponding immunizing peptide, abolished staining of both bands indicating that they represent the seabass BDNF (*data not shown*). In the liver, BDNF was mostly in the mature form (58% of total BDNF) nevertheless, there was also a large amount of proBDNF (42% of total BDNF; Fig. 8A). No statistically significant difference was observed in proBDNF and matBDNF in stressed animal (Fig. 8A, B). Similarly, in the brain, the mature form consisted in 60% of total BDNF and the proBDNF in 40% of total BDNF (Fig. 9B). 30 min of acute stress had no effects on the total amount of BDNF in the brain but induced a highly significant increase in the proBDNF levels and a corresponding significant reduction in mature BDNF (p < 0.01 vs. control, Fig. 9B). The scatter-plot distribution analysis of the two populations showed that in the brain of every animal of the stressed group, the percentage of proBDNF is at least 1 standard deviation (SD) above the mean value of the control group (Fig. 9C). Analogous distribution, but towards lower levels, was also found for mature BDNF (*not shown*). We further calculated if the measurement of the percentage of proBDNF on total BDNF in the brain could represent a predictive test to identify stressed animals. Therefore, we calculated both sensitivity and specificity which are statistical measures of the performance of a binary classification test (see methods). All stressed animals had ratio of proBDNF/totBDNF above 1 SD from the mean value of controls (15/15 stressed are true positive = a, and 0/15 are false negative = c; see methods), hence the sensitivity of the test is 100% while the specificity is 87% due to the presence of two false positives (2/15 controls are false positive = b, thus 13/15 are true negative = d; see methods). The test also had a Positive Predictive Value of 88% and a Negative Predictive Value of 100% (see methods). In sum, this test is a perfect exclusion test, which means that all specimen with proBDNF/totBDNF values below 43% are certainly not stressed. Thus, the brain proBDNF/totBDNF ratio (or its counterpart matBDNF/totBDNF) owing to its remarkable difference between stressed and non-stressed animals represents a highly reliable neurological biomarker capable to detect biological stress in seabass.

## Discussion

This study concerns the first characterization of the BDNF gene in *Dicentrarchus labrax *and its transcriptional, translational and post-translational regulation following acute stress. We found that in the seabass, BDNF is highly expressed in the brain and that the transcripts 1c and 1d from the second promoters cluster are down regulated after acute stress. In addition, we show that acute stress induces a significant increase in the proBDNF levels and a corresponding reduction in mature BDNF suggesting altered regulation of proBDNF proteolytic processing. Finally we show that the proBDNF/totBDNF ratio (or its counterpart matBDNF/totBDNF) is a highly reliable novel quantitative neurological biomarker capable to detect biological stress in fishes with sensitivity 100%, specificity 87%, Positive Predictive Value of 88% and Negative Predictive Value of 100%.

All known vertebrate BDNF genes share a similar multiple exons organization and encode for a pre-pro-protein that is translocated to the endoplasmic reticulum and proteolitically processed to yield the mature protein [52-54]. The *D. labrax *BDNF gene consists of at least five alternative 5'-exons and one 3'-coding exon. For what concern the nomenclature, we have referred to previous studies on zebrafish [55] because mammalian, avian and amphibians BDNF follow a different nomenclature [56]. Analysis of *D. labrax *BDNF transcripts, carried out both in the early developmental stages and in adult tissues, shows that all alternative upstream exons (1β, 1a-1d) are spliced to the protein-coding exon 2. This indicates that *D. labrax *BDNF transcripts structure is similar to other vertebrates. In fact, multiple transcriptional initiation sites and splicing into two-part transcripts can also be found in humans, chimpanzees, dogs, pigs, cats, cows, chicken, frogs, lampreys, zebrafish, and pufferfish [55].

With the exception of the exon 1a (43%), we found strong sequence homology with zebrafish and pufferfish genes for most exons. Furthermore, exon 1β contains 170 nt segment that is highly similar to human exon 1 (75% identity). Three highly conserved segments were found in the seabass BDNF, HCS2 in exon 1a which is present also in exon IIc of mammalian BDNF; HCS1 in exon 1c also found in mammalian exon IV while in the 3'UTR encoded by exon 2, we have found the HCS3 which is also present in the 3'UTR in mammals. These results suggest that mammalian exons I, II, and IV (coding for the the 5' untranslated region) evolved early in the vertebrate radiation and may play a major role in BDNF action, while more recently evolved splice variants including other 5'exons may participate in more specialized functions of BDNF such as, for example, synaptic plasticity. Further research in this direction may allow to test this hypothesis.

The post-hatching developmental analysis indicates that although in different amounts, all *D. labrax *BDNF transcripts, except 1d/2, are well represented at all stages analyzed (day post-hatching 6, 16, 27, 33, 44). On the other hand, the distribution of various BDNF transcripts in adult seabass is tissue specific with all transcripts being most expressed in the brain. The splice form 1c/2 was also expressed, even though at low levels, in all the examined extra nervous tissues (liver, kidney, muscle), while exon 1b/2 transcript was found only in the kidney. Organ-specific expression also holds for most BDNF exons in zebrafish and mammals suggesting conserved transcriptional regulation among the vertebrates [47,52-55].

According to this view, our bioinformatic analysis of *D. labrax *BDNF gene suggests that the region upstream to exon 1c contains two potential responsive elements, belonging to the CRE family. These elements function as responsive elements also in BDNF exon IV of rat cortical neurons [47,55] and may be responsible of the higher expression of the isoform 1c/2 in adult seabass tissue. Of note, a previous study on transcriptional analysis of Zebrafish HCS1 reported that this highly conserved sequence in the 5' exon 1c (and vertebrate exon IV) has properties of a dehancer and, depending on the sequence context, as an enhancer [55].

In the second part of our study we have examined the expression of BDNF transcripts after acute stress caused by water deprivation for 30 minutes. Although no significant difference was found in the total BDNF mRNA levels between stressed and control groups (measured by analysis of the protein-coding exon 2, common to all transcripts), we found a significant decrease in exons 1c and 1d. This finding is consistent with data in rodents in which single immobilization stress induces down regulation of exon IV, omologous to fish exons 1c (they both contain HCS1), due to decreased histone acetylation at this promoter immediately after acute stress [17]. Of note, in a recent study we showed that exons of the second promoter clusters (mammalian exons IV-VII, fish 1c-d), are particularly important for cell survival in response to cellular excytotoxic stress in human neuroblastoma cells [57]. Thus, activation of promoters upstream to these exons might related to a rapid adaptative response to various types of stress.

Western blot analysis showed that in brain, but not in liver, proBDNF content is significantly increased in the stressed samples. Mammalian BDNF transcripts produce the well-known 32 kDa propeptide precursor that is cleaved either to pro28KDa or to the mature 14 kDa BDNF forms by two different proteases [58]. Pro28Kda BDNF peptide is not further processed into the mature 14 kDa BDNF form but it represents a true final proteolytic product generated by a specific Ca^2+^-dependent serine proteinase known as Membrane-Bound Transcription Factor Site-1 protease (MBTFS-1; EC = 3.4.21.112, Alternative names: S1P endopeptidase, Site-1 protease), also known as Subtilisin/kexin-isozyme 1 (SKI-1) [51], while mature 14 KDa BDNF is generated intracellularly by furin [58], or extracellularly by plasmin and matrixmetalloprotease-7 [59]. In contrast, in the seabass we only found two BDNF forms, a proBDNF form corresponding to mammalian pro32KDa precursor and a mature BDNF, while the pro28KDa peptide was absent. Comparison of *D. labrax *BDNF protein with that of rodents and human BDNF, revealed that the mammalian SKI-1 cleavage site at Threonine 57 (Arg-Gly-Leu-Thr↓) is absent in fishes and amphibians and has first emerged in reptilians during vertebrates evolution [54]. Limited proteolysis of one inactive precursor to produce active peptides and proteins is a general mechanism to generate biologically diverse products from a single gene. Here, we provide the first evidence that fishes possess a simplified proteolytic regulation of BDNF and that the pro28KDa proteolytic product, whose function remains yet to be determined, is absent at this stage of vertebrates evolution.

We found that acute stress profoundly alters the relative amount of proBDNF and mature BDNF. Our data are suggestive of a lower proteolytic activity to generate mature BDNF and thus, the uncleaved product is accumulated in the seabass brain, but not in liver, immediately after an acute stress. Although, the mechanisms by which stress can prevent efficient conversion of proBDNF into mature BDNF are presently unknown, several recent studies have pointed out that pro32KDa BDNF has a biological function distinct from that of mature BDNF. Both proBDNF precursor and mature BDNF can be released from neurons [59,60]. While proBDNF binds only to p75 receptor, mature BDNF displays high affinity to TrkB and lower affinity to p75 [61]. Binding of proBDNF to p75 promotes cell death and attenuates synaptic transmission by inducing long term depression [62,63], while mature BDNF sustains long term potentiation and cell survival [59,64,65]. It is therefore conceivable that the shift towards higher proBDNF and lower BDNF level observed after acute stress may have the biological role of attenuating pro-active behavior inducing reduced activity in stressed animals. Stress affects the hormonal response in fish in much the same way it does in higher animals. Stress stimulates the hypothalamus, one of the oldest parts of the brain (in evolutionary terms) and is responsible for controlling the most basic functions such as hunger, thirst, sex drive and, in mammals, body temperature; all functions that are mediated also by BDNF. A reduced behavioral activity may thus represent an adaptive response to dangerous situations represented here by shallow waters, to allow for an immediate energy saving and recovery in preparation for future actions. In this context, it is striking that 100% of animals in our experimental stress group showed >1SD increase in proBDNF levels (and corresponding decrease in mature BDNF). A theoretical, optimal prediction test can achieve 100% sensitivity (i.e. predict all people from the sick group as sick) and 100% specificity (i.e. not predict anyone from the healthy group). Thus, our test performances will make it feasible to screen for stress even in low prevalence populations, particularly where samples are first pooled before testing.

In conclusion, we have determined the structure of *Dicentrarchus labrax *BDNF gene, its expression in neuronal and non neuronal tissues, and we have demonstrated that the proBDNF/totBDNF ratio (or its counterpart matBDNF/totBDNF) is a novel quantitative neurological biomarker capable to detect biological stress in fishes with sensitivity 100%, specificity 87%, Positive Predictive Value of 88% and Negative Predictive Value of 100%.

## Conclusion

The high predictivity of proBDNF/totBDNF ratio for stress in lower vertebrates indicates that processing of BDNF is a central mechanism in adaptation to stress and predicts that a similar regulation of pro/mature BDNF has likely been conserved throughout evolution of vertebrates from fish to man.

## Authors' contributions

CT carried out most of the molecular biology experiments, FR participated in the molecular biology experiments, FDC carried out western blot analysis, GB participated in the western blot analysis, ET designed the western blot experiments and participated to draft the manuscript, GT and MS designed and carried out animal treatment and sacrifice, GB and RG conceived the study, designed and coordinated molecular biology experiments and drafted the manuscript. All authors read and approved the final manuscript.
